# The History and Capabilities of Herbal Simples

**Published:** 1890-01-04

**Authors:** W. T. Fernie


### The History and Capabilities of Herbal Simples.

By W. T. Fernie, M.D.

### II.—A EULOGY, AND SOME ANCIENT AUTHORITIES.

For the sweetest eulogy of our native simples that was ever penned, commend me to a *libellus* indited by Margaretta More—*quindecem annos nata*—in the fifteenth year of her age, at Chelsea, near London, which sets forth in loving, pathetic language the daily doings in the household of Sir Thomas More, her most gracious and martyred father. Erasmus, of learned memory,—the deare little man—is seen by Margaret coming up from the riverside with her father. She "flew upstairs to advertise mother, who was half in and half out of her grogram gown, and who stayed me," says Margaret, "to clasp her owches. Presently they took water bravelie, being rowed by Will and Rupert, who had made themselves spruce enow with nosegays and ribbons, John Harris being seated in the stern and playing the recorder. Landing at Fulham, we had a brave ramble through the meadows. Erasmus, noting the poor children gathering the dandelion and milk thistle for the herb market, was advised to speak of forayn herbs and their uses, bothe for food and medicine. 'For me,' says father, 'there is manie a plant I entertayn in my garden and paddock which the fastidious woulde cast forthe. I like to teache my children the uses of common things; to know, for instance, the uses of the flowers, and weeds that grow in our fields, and hedges. Manie a poor knave's pottage would be improved if he were skilled in the properties of the burdock, and purple orchis, lady's smock, brooklime, and old man's pepper. The roots of wild succorie and water-arrowhead mighte agreeablie change his Lenten diet, and glasswort afford him a pickle for his mouthfulle of salt meat. Then there are cresses and woodsorrel to his breakfast, and saleep for his hot evening mess. For his medicine there is herb twopence, that will cure a hundred ills, camomile to lull a raging tooth, and the juice of buttercup to clear his head by sneezing; vervain cureth ague, and crow-foot affords the least painfulle of blisters; St. Anthony's turnip is an emetic, goosegrass sweetens the blood, woodruffe is good for the liver, and bindweed hath nigh as much virtue as the forayn scammony; pimpernel promoteth laughter, and poppy, sleep. Thyme giveth pleasant dreams, and an ashen branch drives evil spirits from the pillow. As for rosemarie, I let it run alle over my garden walls, not onlie because my bees love it, but because 'tis the herb sacred to remembrance, and therefore to friendship; whence a sprig of it hath a dumb language that maketh it the chosen emblem at our funeral wakes, and in our buriall grounds. Howbeit, I am a schoolboy prating in presence of his master, for here is John Clement at my elbow, who is the best botanist and herbalist of us all.'"

To begin with, the Agrimony is a herbal simple well known to all country folk, and abounding throughout England, in its fields and woods, as a popular domestic medicine. It is called Agrimonia Eupatoria, and belongs to the rose tribe of plants. The first of these names is from the Greek—*argemone*—shining—as applied to a plant able to cure cataract of the eye; the second is also from a Greek word, *hepatos*, belonging to the liver, and it indicates the reputed use of the herb for curing diseases of that organ. The agrimony blossoms from June to September, with small yellow flowers, which smell like apricots, and sit close on slender spikes, a foot high, called by rustics church steeples. Chemists have determined that the agrimony possesses a particular volatile oil, and yields nearly five per cent. of tannin, so that its popular use for gargles and as an astringent application to wounds and sores is well justified. I cannot find that the plant really owns any qualities for acting medicinally on the liver. More probably the yellow colour of its flowers, which, with the root, supply a dye of a bright nankeen hue, first gave it a reputation in bilious disorders, according to the doctrine of signatures, because the bile is also yellow. This doctrine was much in vogue with our forefathers; it supposed that Nature advertises her remedial intentions by the colours which she assigns to the blossoms, and juices of plants as pointing to the maladies for which she means them to be used. Likewise it was thought that something in the external aspect of certain plants revealed their special curative virtues. Thus it was believed the hard stony seeds of Gromwell lithospermum were good for gravel, the knotty tubers of scrophularia for scrofulous glands, the scaly husk of the scabious for leprosy of the skin, the spotted leaves of the lungwort for consumption, and the saxifrage, because it finds its way through rocks, for breaking up stone in the bladder.

"Though sin and Sathan have plunged mankinde into an ocean of infirmities, yet the mercy of God maketh grasse to grow upon the mountaines, and herbes for the use of man, and hath not only stamped upon them a distinct forme, but hath also given them particular signatures whereby a man may read, even in legible characters, the use of them." It is certainly remarkable that though this fanciful theory has been long since exploded, yet doctors of to-day still prescribe several yellow medicines for a disordered liver, as the greater celandine, with its ochre-like juice—the dandelion, the yellow barberry, the golden seal, hydrastis, and yellow metallic gold.

By pouring a pint of boiling water on a handful of agrimony—the stem, flowers, and leaves—an excellent gargle may be made for a relaxed throat; and a teacupful of the same infusion may be taken, when cold, three or four times a day, for simple looseness of the bowels, also for some losses of blood. In France agrimony-tea is taken as a beverage at table. Pliny calls it a herb of princely authority; and Gerard says "a decoction of its leaves is good for them that have naughty livers." The plant was at one time included in the *London Materia Medica*, and was of old one of the famous vulnerary herbs. It formed an ingredient of the genuine arquebusade water. Because its seed vessels cling by the hooked ends of their stiff hairs to any person or animal coming into contact with the plant, it is often uamed cockleburr, or sticklewort. A curious fact about the agrimony is that, whilst the rest of the rose tribe to which it belongs have marched steadily onwards in the race of evolution, from the diminutive cinquefoil of the hedgerow to the berries, the roses (wild and cultivated), the plum-trees, pear-trees, and apple-trees, this plant has stood still in the struggle, and retains its primitive original features. It yet scarcely exceeds a foot in height, and bears inconspicuous yellow flowers which secrete no honey. Moreover, so as to attract the visits of fertilising insects, these flowers are huddled together, like a humble republic, on a common terminal spike. The plant stands like an obsolete milestone on an old superannuated Roman highway now superseded by the swift and relentless railroad. Maybe some future botanical Pickwick will yet hail the isolated natural production, and will marvel at its primitive characteristics with such exultation as filled the large heart of that literary hero when he unearthed the village stone bearing that immortal inscription—Bil-stumpshismark! or, for ages yet to come the changeless herb may survive to preach the fundamental text, that whilst

"From a shapeless albuminous dot
We mortals our beings derive,
In the mud of some Cambrian spot
Did our earliest ancestor dive;
He could split himself up into five,
Or roll himself round like a ball;
So the fittest will always survive,
Whilst the weakest will go to the wall."

